# Health-Related Lifestyle Behaviors among Male and Female Rural-to-Urban Migrant Workers in Shanghai, China

**DOI:** 10.1371/journal.pone.0117946

**Published:** 2015-02-24

**Authors:** Hua Yang, Fang He, Tianhao Wang, Yao Liu, Yao Shen, Jian Gong, Wei Dai, Jing Zhou, Jie Gu, Yimin Tu, Tianying Wang, Lei Shen, Yumiao Wu, Xiuping Xia, Donghao Xu, Zhigang Pan, Shanzhu Zhu

**Affiliations:** 1 Department of General Practice, Zhongshan Hospital of Fudan University, Shanghai, China; 2 Department of General Practice, Xinjing Community Health Service Center, Shanghai, China; 3 Department of General Practice, Yinhang Community Health Service Center, Shanghai, China; 4 Department of General Practice, Sanlin Community Health Service Center, Shanghai, China; 5 Department of Preventive Medicine, Huaxin Community Health Service center, Shanghai, China; 6 Department of General Practice, Changzheng Community Health Service Center, Shanghai, China; 7 Department of General Practice, Huangdu Community Health Service Center, Anting Town, Shanghai, China; 8 Department of General Practice, Caohejing Community Health Service center, Shanghai, China; Leibniz Institute for Prevention Research and Epidemiology (BIPS), GERMANY

## Abstract

**Background:**

Lifestyle behaviors significantly impact health, yet remain poorly defined in Chinese rural-to-urban migrants.

**Methods:**

In a cross-sectional study of health-related behaviors of 5484 rural-to-urban migrants who had worked in Shanghai for at least six months, we assessed the contribution of demographics and physical and mental health to lifestyle behaviors in male and female participants by multiple stepwise cumulative odds logistic regression.

**Results:**

Respondents were 51.3% male. 9.9% exhibited abnormal blood pressure; 27.0% were overweight or obese; 11.2% reported abnormal mental health; 36.9% reported healthy lifestyle. Multiple stepwise cumulative odds logistic regression indicated that men working in manufacturing reported less unhealthy lifestyle than those in hospitality (cumulative odds ratio (COR) = 1.806, 95%CI 1.275–2.559) or recreation/leisure (COR = 3.248, 95%CI 2.379–4.435); and women working in manufacturing and construction reported less unhealthy lifestyle than those in all other sectors. Unhealthy lifestyle was associated with small workplaces for men (COR = 1.422, 95%CI 1.154–1.752), working more than 8 or 11 hours per day for women and men, respectively, and earning over 3500 RMB in women (COR = 1.618, 95%CI 1.137–2.303). Single women and women who had previously resided in three or more cities were more likely to report unhealthy lifestyle (COR = 2.023, 95%CI 1.664–2.461, and COR = 1.311, 95%CI 1.072–1.602, respectively). Abnormal mental status was also correlated with unhealthy lifestyle in men (COR = 3.105, 95%CI 2.454–3.930) and women (COR = 2.566, 95%CI 2.024–3.252).

**Conclusions:**

There were different risk factors of unhealthy lifestyle score in male and female rural-to-urban migrants, especially in number of cities experienced, salary, marital status, work place scale. Several demographic groups: employment sectors (e.g. hospitality and recreation/leisure), working conditions (e.g. long hours) and abnormal mental status were associated with unhealthy lifestyle behaviors in Chinese rural-to-urban migrants, and health interventions should be targeted to these groups.

## Introduction

Health-related lifestyle behaviors are important determinants of disease and mortality fundamental to public health [1–5]. Lifestyle behaviors that have been reported to impact health include cigarette smoking; alcohol consumption; physical activity, diet [1, 6–9], and health status inequalities between different socioeconomic groups [10]. Some studies have designed health scores based on assessment of several health-related lifestyle behaviors [11–15]. To date, however, these approaches have rarely been applied to assess the health-related lifestyle behaviors of migrant workers in China [16]. Whilst the prevalence of infectious diseases, mental health, quality of life and smoking in this group has received recent attention [17–21]; comprehensive assessment of a variety of lifestyle behaviors has not yet been reported in this population.

Shanghai, one of the largest economic centers in China, attracts 6 million migrants annually, and currently these migrants account for about a quarter of the total population of Shanghai [22]. The Shanghai Bureau of Statistics estimates that 80% of migrant workers are married, with an average age of 38. 72% have not completed high school; 78.4% live in rented accommodation; 22% work overtime daily or frequently; and 80% have employee’s insurance or rural cooperative medical care insurance [23–26]. Insight into the lifestyle behaviors of migrant workers would facilitate the development of targeted primary prevention strategies.

In this cross-sectional study, we used a lifestyle score based on six behaviors to assess the health related lifestyle behaviors of male and female rural-to-urban migrant workers in Shanghai. Furthermore, we explored the association between lifestyle scores and contributing factors such as employment, migratory history, marital status, salary and mental status in male and female rural-to-urban migrant workers.

## Materials and Methods

### Study population

In this cross-sectional study, conducted between August and October 2012 in Shanghai, we employed a proportionally stratified multistage cluster random sampling procedure to recruit participants. We chose to recruit the subjects from four of the eight central and three of the eight fringing districts (Xuhui, Putuo, Changning, Yangpu, Pudong, Jiading, and Qingpu), respectively, in each of which one community health service center was randomly selected, hence Caohejing, Changzheng, Xinjing, Yinhang, Sanlin, Huangdu, and Huaxin Community Centers. Based on available government statistics regarding migrant occupations in Shanghai [27], migrant workers were sampled from six occupations: 1) manufacturing, 2) construction, 3) hospitality, 4) domestic service, 5) small business and 6) recreation/leisure. In selected health service centers, cluster random sampling was conducted according to the workplace. Workplaces at which more than 50% of employees were migrants were eligible. All the eligible workplaces within the service area were classified in three strata (large, ≥500 employees; moderate, 100–500 employees; small, ≤100 employees). According to the proportion of migrant workers in each sector reported in the government statistics [27], in large workplaces no more than 200 migrants were cluster sampled according to workgroup. In moderately sized workplaces no more than 150 migrants were sampled. In small workplaces all the migrants were sampled.

All the employers (or managers) consented to conduction of the surveys on their premises. From each community health center, the general practitioners and nurses approached the rural-to-urban migrants (with rural household registration that had migrated from rural to urban areas for the purpose of employment) at the sampling sites, who were aged 18–65 years with a residency of at least 6 months. Written informed consent was obtained from all volunteers, and the survey questionnaire was individually administered under supervision. Considering the prevalence of smoking, alcohol consumption and mental disorders, and 10% questionnaire failure rate, we calculated the required sample size to be 5800. For each volunteer, blood pressure was measured on the spot only once (normal blood pressure defined as <140/90mmHg and abnormal blood pressure as ≥140/90mmHg). Assistance was provided to those who had difficulty completing the questionnaire primarily due to their limited years of education. The confidentiality of responses was ensured during the survey. All completed questionnaires were exclusively accessible to the investigators and researchers, who were regulated by the rules of confidentiality. The study protocol was approved by the Ethics Committee, Zhongshan Hospital of Fudan University (B2013–138).

### Survey questionnaire

To assess the understandability and feasibility in the target population, the questionnaire was pilot-tested at the 7 community health service centers with migrants of different ages and education levels. The size of questionnaire and wording of the questions were revised via discussion prior to launching data collection. It took approximately 45 min to complete the 13-page survey, composed of 4 sections as follows.

### Socio-demographic characteristics

Socio-demographic characteristics including age, gender, occupation, workplace scale, educational attainment, marital status, accompanying child/children, salary, years of residency in Shanghai, number of cities resided in, hours worked per day, days worked per week, and type of residence were recorded.

Income was categorized as < RBM 1500, RBM 1500–2500, RBM 2500–3500 and ≥RBM 3500. In 2012, the minimum income standard in Shanghai was RMB 1450 per month [28], and the average income of migrant workers in a municipality like Shanghai, Beijing, was RMB 2561 per month [28]. Shanghai urban residents per capita disposable income was RMB 3350 per month [29]. Thus our categories were designed to reflect the fact that an income below RMB1500 per month indicates an individual below the poverty level, and an income above RBM3500 per month is above average level.

### Mental health

To assess mental health we employed a Chinese version of the Symptom Checklist-90-Revised (SCL-90-R)[30], a self-reported mental health questionnaire. An SCL-90 score ≤160 indicates normal mental health, and a SCL-90 score >160 abnormal mental health [31]. These thresholds are widely used in Chinese research [32,33].

### Physical health

BMI was calculated from self-reported height and weight according to the Chinese criteria [34], a BMI<18.5 indicating underweight, a BMI between 18.5 and 24 normal weight, and a BMI ≥24 overweight or obesity. A history of one or more of the following chronic diseases was recorded: hypertension, ischemic heart disease, diabetes, chronic obstructive pulmonary disease, asthma, renal dysfunction, abnormal liver function, rheumatoid arthritis, osteoarthritis, or mental illness.

### Lifestyle behaviors

In the fourth section were recorded six lifestyle behaviors as smoking status, alcohol consumption, sleep duration and quality of sleep, breakfast patterns, frequency of regular meals and frequency of fruit and vegetable consumption.

Lifestyle behaviors that have been reported to impact health included cigarette smoking, alcohol consumption, physical activity, diet habits and sleep duration [1, 6–9]. However, as rural-to-urban migrants mostly engaged in physical labor, likely fulfilling their requirements for physical activity, we did not survey physical activity. To assess participant diet we surveyed the frequency of fruit and vegetable consumption, frequency of regular meals and frequency of breakfast, which have previously been established to be relevant to some diseases like chronic gastritis or cholecystitis [3, 6, 11]

The participants were classified into three categories, current smokers (with a history of smoking in the past 30 days), previous smokers (≥100 cigarettes in their lifetime) or nonsmokers, according to previously published analyses [35,36]. For multivariate analysis, we dichotomized smoking status as current versus previous smokers/nonsmokers, with one point allocated to current smokers.

Participants were asked to categorize alcohol consumption according to the Alcohol Use Disorders Identification Test-Consumption questionnaire (AUDIT-C) [37], in response to the questions “How often do you have a drink containing alcohol?”, “How many drinks containing alcohol do you have on a typical day when you are drinking?” and “How often do you have six or more drinks on one occasion?” The response to each question is scored 0 to 4 points to yield a total of 12 points. Scores ≥4 in men or ≥3 in women indicate positive screens for hazardous alcohols use. We dichotomized this variable as nonhazardous alcohol use versus hazardous alcohol use, and allocated 1 point for the latter [38, 39].

Participants reported the average number of hours per night spent sleeping over the previous month, and 1 point was allocated for an average of <7 hours of sleep or >9 hours of sleep, as previously established as a risk factors for health [40]. Additionally, participants reported their quality of sleep over the previous month as good, fair, poor or very poor.

Participants described their frequency of having breakfast as almost daily, 3–4 times per week, 1–2 per week, or rarely/never, and for multivariate analyses, we dichotomized all responses into “almost daily” *vs*. “no daily breakfast”. Based on the dietary guidelines for Chinese residents [41], participants described their frequency of fruit or vegetable consumption as almost daily, 1–2 per week, 1–2 per month, or rarely/never. Additionally, they described the frequency of having regular meals as almost daily, 1–2 per week, 1–2 per month or rarely/never. We dichotomized both “consumption of fruits and vegetables” and “frequency of having regular meals” into “almost daily” *vs*. “not daily” for multivariate analyses.

Based on these six factors, we used a lifestyle score similar to that described in the previous studies [11–15, 42–44]. To each factor we allocated 0 or 1 points. An overall score, ranging from 0 to 6, indicated one of three subcategories: healthy (0 point), relatively healthy (1–2 points) or unhealthy (3–6 points).

### Statistical analysis

The data obtained during the current study was submitted to Dryad according to the PLOS data policy. Statistical analysis was carried out using SPSS software, version 17.0 (SPSS Inc, Chicago USA) and SAS software, version 9.2 (SAS Institute, Cary, NC).

For socio-demographic characteristics and health status, we calculated frequencies or mean values and standard deviations by gender. The differences between male and female migrants were analyzed using Chi-square test for frequencies, and un-paired *t* test for mean values.

A multiple stepwise cumulative odds logistic regression model was used to assess the association between unhealthy lifestyle score and multiple risk factors by gender. Healthy lifestyle level was used as reference in this model. The independent variables were age, occupation, workplace scale, educational attainment, marital status, accompanying child/children, salary, years of residency, cities resided in, hours worked per day, days worked per week, type of residence, self-reported health status, chronic disease, blood pressure, BMI, and mental status. The significance level for entering effect was *P*<0.05 and removing effect was *P*≥0.05. Cumulative odds ratios (COR) and 95% confidence intervals (CIs) were calculated. A 2-tailed alpha with *P*<0.05 was considered statistically significant for all analyses.

## Results

### Socio-demographic characteristics of participants

Based on the analysis of the complete questionnaire responses of 5484 (93.7%) without missing data from the total number of 5855 participants approached (Table 1), it was found that 51.3% of participants were male; 48.2% were new-generation migrant workers, who were born into farming families in rural areas after 1980 and migrated to urban areas after graduating from school [45]; 50.9% completed junior high school and 24.1% finished high school; 73.3% were married and 70.0% had children. 77.1% earned RMB 1500–3500 per month, a salary range of the poverty level to urban residents per capita disposable income in Shanghai [28,29], but 68.7% worked more than 5 days a week, and 51.5% worked more than eight hours per day. 53.5% rented a room as a family or with others; and 28.4% lived in collective dormitories (Table 1).

**Table 1:** Socio-demographic characteristics of rural-to-urban migrant population by gender.

Variables	Total (n = 5484)	Male (n = 2811)	Female (n = 2673)	*P*-value*
Age (years), mean±SD	34.3±10.5	35.4±10.9	33.2±10.0	<0.001^Δ^
Age (years), n (%)				
≤32	2645 (48.2)	1237 (44.0)	1408 (52.7)	<0.001^#^
>32	2839 (51.8)	1574 (56.0)	1265 (47.3)	
Occupation, n (%)				
Manufacturing	2513 (45.8)	1406 (50.0)	1107 (41.4)	<0.001^#^
Construction	769 (14.0)	677 (24.1)	92 (3.4)	
Hospitality	385 (7.0)	148 (5.3)	237 (8.9)	
Domestic service	574 (10.5)	144 (5.1)	430 (16.1)	
Small business	635 (11.6)	232 (8.3)	403 (15.1)	
Recreation/leisure	608 (11.1)	204 (7.3)	404 (15.1)	
Workplace scale, n (%)				
Large	1500(27.4)	851(30.3)	649(24.3)	<0.001^#^
Moderate	1942(35.4)	1101 (39.2)	841 (31.5)	
Small	2042(37.2)	859 (30.6)	1183 (44.3)	
Educational attainment, n (%)				
Elementary or lower	1004 (18.3)	393 (14.0)	611 (22.9)	<0.001^#^
Junior high school	2789 (50.9)	1409 (50.1)	1380 (51.6)	
High school	1320 (24.1)	777 (27.6)	543 (20.3)	
College	371 (6.8)	232 (8.3)	139 (5.2)	
Marital status, n (%)				
Married	4019 (73.3)	2042 (72.6)	1977 (74.0)	0.179^#^
Single	1331 (24.3)	700 (24.9)	631 (23.6)	
Cohabitating	86 (1.6)	39 (1.4)	47 (1.8)	
Divorced or widowed	48 (0.9)	30 (1.1)	18 (0.7)	
Accompanying children, n (%)				
No children	1645 (30.0)	849 (30.2)	796 (29.8)	<0.001^#^
Cohabiting children	1846 (33.7)	878 (31.2)	968 (36.2)	
Children residing elsewhere	1993 (36.3)	1084 (38.6)	909 (34.0)	
Salary, n (%)				
<1500 RBM	485 (8.8)	168 (6.0)	317 (11.9)	<0.001^#^
1500–2500 RBM	2528 (46.1)	1036 (36.9)	1492 (55.8)	
2500–3500 RBM	1699 (31.0)	1071 (38.1)	628 (23.5)	
≥3500 RBM	772 (14.1)	536 (19.1)	236 (8.8)	
Years of residency, n (%)				
<1	735 (13.4)	365 (13.0)	370 (13.8)	<0.001^#^
1–5	2432 (44.3)	1165 (41.4)	1267 (47.4)	
≥5	2317 (42.3)	1281 (45.6)	1036 (38.8)	
Number of cities resided in, n (%)				
1–2	4066 (74.1)	1859 (66.1)	2207 (82.6)	<0.001^#^
≥3	1418 (25.9)	952 (33.9)	464 (17.4)	
Daily working hours, n (%)				
<8	80 (1.5)	18 (0.6)	62 (2.3)	0.933^#^
= 8	2583 (47.1)	1273 (45.3)	1310 (49.0)	
8–11	1681 (30.7)	1066 (37.9)	615 (23.0)	
≥11	1140 (20.8)	454 (16.2)	686 (25.7)	
Weekly working days, n (%)				
≤4	156 (2.8)	57 (2.0)	99 (3.7)	<0.001^#^
5	1564(28.5)	805(28.6)	759 (28.4)	
6	2247 (41.0)	1046 (37.2)	1201 (44.9)	
7	1517 (27.7)	903 (32.1)	614 (23.0)	
Type of residence, n (%)				
Collective dormitory	1555 (28.4)	1040 (37.0)	515 (19.3)	<0.001^#^
Renting with others	636 (11.6)	324 (11.5)	312 (11.7)	
Renting as a family	2297 (41.9)	897 (31.9)	1400 (52.4)	
Renting alone	754 (13.7)	430 (15.3)	324 (12.1)	
Owning a living place	242 (4.4)	120(4.3)	122 (4.6)	

*Note*: n: number; SD: standard deviation;

*: comparing male and female;

^Δ^: using un-paired *t* test;

^#^: using Chi-square test.

On average, the male migrants were older than female migrants (*P*<0.05). Significant differences were observed between the demographic characteristics of male and female migrants. A higher proportion of male migrants had completed high school or college and earned over RMB 2500 per month (*P*<0.05). Occupation, workplace scale, accompanying child/children, years of residency in Shanghai, number of cities resided in, days worked per week, and type of residence also differed significantly between male and female participants (*P*<0.05). Characteristics which did not differ significantly between male and female participants included marital status and hours worked per day (Table 1).

### Health status of migrant population

The vast majority of migrants self-reported good or fair health (98.9%), and only 8.2% reported chronic diseases. However, 9.9% exhibited abnormal blood pressure; 27.0% were overweight or obese; 11.2% had scores indicating abnormal mental health on the SCL-90-R; and on assessment of reported health-related behaviors, only 36.9% were classified as having a healthy lifestyle (Table 2).

**Table 2:** Physical and mental status and lifestyle behavior of rural-to-urban migrants by gender.

Variables	Total (n = 5484)	Male (n = 2811)	Female (n = 2673)	*P*-value*
**Health status**				
Self-reported health status, n (%)				
Good	4278 (78.0)	2194 (78.1)	2084 (78.0)	0.966^#^
Fair	1148 (20.9)	585 (20.8)	563 (21.1)	
Poor	58 (1.1)	32 (1.1)	26 (1.0)	
Chronic diseases, n (%)	449 (8.2)	266 (9.5)	183 (6.8)	<0.001^#^
Abnormal blood pressure, n (%)	545 (9.9)	401(14.3)	144 (5.4)	<0.001^#^
BMI				
Underweight	346 (6.3)	114 (4.1)	232 (8.7)	<0.001^#^
Normal	3655 (66.6)	1775 (63.1)	1880 (70.3)	
Overweight/obesity	1483 (27.0)	922 (32.8)	561 (21.0)	
**Mental state**, n (%)				
Normal	4870 (88.8)	2501 (89.0)	2369 (88.6)	0.686^#^
Abnormal	614 (11.2)	310 (11.0)	304 (11.4)	
**Lifestyle behaviors**				
**Smoking status, n (%)**				
Previous smoker/ never smoked	4100 (74.8)	1489 (53.0)	2611 (97.7)	<0.001^#^
Current smoker	1384 (25.2)	1322 (47.0)	62 (2.3)	
**Alcohol consumption, n (%)**				
Nonhazardous alcohol use	4435 (80.9)	1979 (70.4)	2456 (91.9)	<0.001^#^
Hazardous alcohol use	1049 (19.1)	832 (29.6)	217 (8.1)	
**Sleep, n (%)**				
7–9 hours / night	4273 (77.9)	2166 (77.1)	2107 (78.8)	0.114^#^
<7 or >9 hours / night	1211 (22.1)	645 (22.9)	566 (21.2)	
Good or fair quality sleep	4815 (87.8)	2474 (88.0)	2341 (87.6)	0.625^#^
Poor or very poor quality sleep	669 (12.2)	337 (12.0)	332 (12.4)	
**Diet, n (%)**				
Almost daily breakfast	4458 (81.3)	2302 (81.9)	2156 (80.7)	0.241^#^
Almost daily fruit & vegetable consumption	4250 (77.5)	2101 (74.7)	2149 (80.4)	<0.001^#^
Almost daily regular meals	4642 (84.6)	2398 (85.3)	2244 (84.0)	0.613^#^
**Lifestyle score, n (%)**				
Healthy	2026 (36.9)	640 (22.8)	1386 (51.9)	<0.001^#^
Relatively healthy	2545 (46.4)	1545 (55.0)	1000 (37.4)	
Unhealthy	913 (16.6)	626 (22.3)	287 (10.7)	

*Note*: n, number; SD, standard deviation;

* male in comparison to female;

^#^ using Chi-square test.

Although self-reported health or mental health did not differ significantly between male and female participants, male participants reported significantly higher rates of chronic disease (9.5%), abnormal blood pressure (14.3%), abnormal BMI (36.9%), current smoking (47.0%), and hazardous alcohol consumption (29.6%), than female participants (6.8%, 5.4%, 29.7%, 2.3% and 8.1%, respectively) (*P*<0.05). Female participants reported a higher frequency of fruit and vegetable consumption and higher healthy lifestyle scores (51.9% healthy, 37.4% relatively healthy, 10.7 unhealthy) than male participants (22.8% healthy, 55.0% relatively healthy, 22.3% unhealthy) (*P*<0.05) (Table 2).

### Association between lifestyle score and socio-demographic characteristics and physical and mental status

The results from the multiple stepwise cumulative odds logistic regression analysis (Table 3) were as follows:

Male participants working in hospitality (COR = 1.806, 95%CI 1.275–2.559, *P*<0.001) or recreation/leisure (COR = 3.248, 95%CI 2.379–4.435, *P*<0.001) were more likely to report an unhealthy lifestyle than those working in manufacturing. Female participants working in hospitality (COR = 1.675, 95% CI 1.263–2.222, *P*<0.001), domestic service (COR = 1.657, 1.314–2.090, *P*<0.001), small businesses (COR = 1.556, 95%CI 1.222–1.981, *P*<0.001) or recreation/leisure (COR = 5.563, 95%CI 4.268–7.250, *P*<0.001) were also more likely to report an unhealthy lifestyle than those working in manufacturing. Men working at small workplaces were more likely to report an unhealthy lifestyle than those working at a large workplaces (COR = 1.422, 95%CI 1.154–1.752, *P*<0.001).

**Table 3:** Multiple stepwise cumulative odds logistic regression of lifestyle score with socio-demographic characteristics and physical and mental status by gender.

Independent Variables	Male			Female		
	COR	95%CI	*P*	COR	95%CI	*P*
Occupation						
Manufacturing	1.0			1.0		
Construction	1.216	0.992–1.489	0.059	1.043	0.669–1.627	0.853
Hospitality	1.806	1.275–2.559	<0.001	1.675	1.263–2.222	<0.001
Domestic service	0.849	0.604–1.193	0.344	1.657	1.314–2.090	<0.001
Small business	1.183	0.891–1.570	0.244	1.556	1.222–1.981	<0.001
Recreation/leisure	3.248	2.379–4.435	<0.001	5.563	4.268–7.250	<0.001
Work place scale						
Large	1.0			-	-	-
Moderate	0.965	0.802–1.161	0.702	-	-	-
Small	1.422	1.154–1.752	<0.001	-	-	-
Marital status						
Married	-	-	-	1.0		
Single	-	-	-	2.023	1.664–2.461	<0.001
Cohabitating	-	-	-	1.564	0.873–2.803	0.133
Divorced or widowed	-	-	-	1.533	0.621–3.784	0.355
Salary						
<1500 RMB	-	-	-	1.0		
1500–2500 RMB	-	-	-	0.817	0.638–1.046	0.109
2500–3500 RMB	-	-	-	0.822	0.622–1.085	0.166
≥3500 RMB	-	-	-	1.618	1.137–2.303	0.008
Number of cities experienced						
1–2	-	-	-	1.0		
≥3	-	-	-	1.311	1.072–1.602	0.008
Hours worked per day						
<8	0.952	0.384–2.359	0.915	1.296	0.781–2.151	0.316
= 8	1.0			1.0		
8–11	1.074	0.907–1.273	0.406	1.426	1.172–1.734	<0.001
≥11	1.533	1.238–1.898	<0.001	1.287	1.055–1.569	0.013
Mental status						
Normal	1.0			1.0		
Abnormal	3.105	2.454–3.930	<0.001	2.566	2.024–3.252	<0.001

*Note*: COR: cumulative odds ratio; 95% CI: 95% Confidence Interval.

Men that worked 11 or more hours per day were more likely to report an unhealthy lifestyle than those that worked for eight hours per day (COR = 1.533, 95%CI 1.238–1.898, *P*<0.001), and in comparison to women that worked for eight hours per day, women that worked for 8–11 (COR = 1.426, 95%CI 1.172–1.734, *P*<0.001) or 11 or more hours per day (COR = 1.287, 95%CI 1.055–1.569, *P* = 0.013) were more likely to report an unhealthy lifestyle.

In comparison to women earning a salary under 1500 RMB, those who earned over 3500 RMB were more likely to report an unhealthy lifestyle (COR = 1.618, 95%CI 1.137–2.303, *P* = 0.008). Single women were more likely to report an unhealthy lifestyle than married women (COR = 2.023, 95%CI 1.664–2.461, *P*<0.001), and women who had previously resided in three or more cities were more likely to have an unhealthy lifestyle than those who had resided in only one or two cities (COR = 1.311, 95%CI 1.072–1.602, *P* = 0.008). Abnormal mental status was also correlated with an unhealthy lifestyle in men (COR = 3.105, 95%CI 2.454–3.930, *P*<0.001) and women (OR = 2.566, 95%CI 2.024–3.252, *P*<0.001).

## Discussion

This large-scale cross-sectional study of migrant workers was conducted in the booming city of Shanghai, with the demographic characteristics, the physical and mental status of male and female rural-to-urban migrants examined and the aspects of their lifestyle assessed so as to determine what characteristics were associated with unhealthy lifestyle behaviors. In our sample less than 10% of respondents reported salaries below the poverty level in Shanghai [28], but the majority worked for more than five days per week, and roughly half worked for more than eight hours per day. Whilst the vast majority of migrants self-reported good or fair health, and few reported chronic diseases, on assessment of reported health-related behaviors, only 36.9% were classified as having a healthy lifestyle.

We observed a lower rate of abnormal blood pressure in this study than in the previous report on the prevalence of hypertension among rural-to-urban migrant workers in Hangzhou, a city in Eastern China [46], and in the general Chinese populations [47]. This discrepancy can be explained by the younger age of the participants (34.3 years on average), who represented an age group in which the incidence of hypertension is typically lower [48], as well as by the possibility of including those who were on medication for hypertension, as indicated by 8.2% of the respondents who reported a chronic medical condition.

The fraction of our overweight or obese samples was lower than that in the national population [49]. Previous studies have suggested that those residing in the rural areas are less likely to be obese, for they tend to engage in manual labor, but that the risk of obesity increases with the years of residence in a city, even for those engaged in urban manual labor as their traditional diets may be replaced with the more prevalent high calorie foods [50,51]. As the majority of our participants would continue to live in the city, it is possible that their BMI would increase.

Previous reports of the mental health of migrants have produced conflicting results, indicated that certain population are at a higher risk for mental illness than the general population [19,21,52,53], whilst others are at a lower risk [18]. Our results support the latter conclusion. Although rural-to-urban migrants may experience a lower quality of life than their urban counterparts [17,20], our survey participants had resided in Shanghai for at least 6 months, and may represent a more settled population than those in previous investigations.

We found that approximately half of the participants reported one or two unhealthy lifestyle behaviors, 16.6% reporting more than two. Although self-reported mental and physical health did not differ significantly between the genders, the male migrants were considerably more likely to report a chronic health condition than the females, showing a significantly higher fraction than the female migrants in terms of abnormal BMI and elevated blood pressure and presenting a much unhealthier lifestyle. The males reported significantly more unhealthy behaviors than the females, except sleep duration and quality, breakfast pattern and irregular meals. Additionally, the males showed higher scores for unhealthy lifestyle than the females, mainly because smoking and drinking were observed to be significantly more common in the males than in the females, as previously reported [54–57].

For men and women, those who worked in recreation/leisure; worked eleven or more than eleven hours per day; or self-reported abnormal mental status, were prone to unhealthy behaviors. Those who worked in recreation/leisure were significantly more prone to unhealthy lifestyle behaviors than those who worked in other sectors, presumably because of job instability and irregular working hours. Those who worked 8 hours per day, as required by the law, reported fewer unhealthy lifestyle behaviors than those who worked more than eleven hours per day, exceeding the overtime per day in law, which served as a marker of a higher work load. Mental status was another important influential factor, and abnormal mental status was a strong negative factor of healthy lifestyle. Jerzy *et al*. reported that subjects with depressive symptoms reported unhealthier lifestyle behaviors in comparison to healthy individuals, for those with worse mental health may be less capable of maintaining a healthy lifestyle [58].

The sex differential of the association between lifestyle score and socio-demographic characteristics and mental status was particularly marked for workplace scale, marital status, salary and number of cities resided in. Men working at small workplaces were more likely to report an unhealthy lifestyle than those working at large workplaces, which could also be attributed to the lower employment stability and reliability of smaller companies. Mechanisms underpinning many of these observed trends are unknown. Particularly interesting was the observation that, in comparison to women earning a salary under 1500 RMB, those who earned over 3500 RMB, representing above urban residents per capita disposable income in Shanghai [28,29] were more likely to report an unhealthy lifestyle. Further studies in this population will be necessary to further elucidate the reasons for these differences. Single women were more likely to report an unhealthy lifestyle than married women, and women who had previously resided in three or more cities were more likely to have an unhealthy lifestyle than those who had resided in only one or two cities, suggesting an unstable home life may contribute to unhealthy lifestyle in women.

Interventions in multiple lifestyle behaviors were reported to be recognized as an effective method to enhance health and reduce health care costs [59]. As indicated by the findings in this study, the need to promote healthy lifestyle behaviors among the migrants is urgent, for this population has limited experience with local healthcare facilities, a limited social network and limited education. Migrants require specific, tailored interventions, as indicated by some factors associated with unhealthy lifestyle, particularly those employed in the recreation/leisure sector, and those women who were single. Potential intervention strategies must be tested in each individual targeted population, as they are sufficiently unique, with characteristics such as younger age, few medical concerns, ignorance to their unhealthy lifestyle behaviors, and limited access to health knowledge and education due to their limited educational background, harsh survival environment, and underprivileged conditions, accompanied by depressed self-esteem and antipathy to the interventions (i.e. public education on smoking cessation) [60–63].

Health interventions may be integrated with the migrant workers’ favorable union of the same origin, and tailoring interventions to specific worksites may represent a viable method for promotion of healthy lifestyles, addressing the specific needs of each group. General practitioners and nurses must maintain a rapport with this population in the local communities, so regular educational information can be delivered to them, and self-assessment of health lifestyle behaviors can be performed in accordance with the findings derived from this study.

In addition, the government should take the responsibility of addressing the health inequalities among migrant workers and take a holistic approach to health promotion and illness prevention strategies for migrant workers.

### Limitations

Lifestyle data was not collected from non-migrant Shanghainese residents, so the lifestyles of native residents and migrant workers could not be directly compared. Self-reported lifestyle information and physician-diagnosed chronic diseases may have resulted in some misclassification, and information bias. Additionally, this study was cross-sectional in design; therefore, only association, rather than causation, could be evaluated, and longitudinal studies will be necessary better elucidate the cause-effect relationships of risk factors and health in this population. Further research into the health and behavior of migrant workers will be required to highlight the healthcare needs of this population.

## References

[1] Centers for Disease Control and Prevention (2013) Chronic diseases and health promotion. Available: http://www.cdc.gov/chronicdisease/overview/index.htm.

[2] Yang ZY, Yang Z, Zhu L, Qiu C (2011) Human behaviors determine health: strategic thoughts on the prevention of chronic non-communicable diseases in China. Int J Behavioral Medicine 18: 295–301. 10.1007/s12529-011-9187-0 21866410

[3] Heidemann C, Schulze MB, Franco OH, van Dam RM, Mantzoros CS, et al (2008) Dietary patterns and risk of mortality from cardiovascular disease, cancer, and all causes in a prospective cohort of women. Circulation 118: 230–237. 10.1161/CIRCULATIONAHA.108.771881 18574045 PMC2748772

[4] Carlsson AC, Theobald H, Wandell PE (2010) Health factors and longevity in men and women: a 26-year follow-up study. European journal of epidemiology 25: 547–551. 10.1007/s10654-010-9472-2 20623324

[5] Mozaffarian D, Kamineni A, Carnethon M, Djousse L, Mukamal KJ, et al (2009) Lifestyle risk factors and new-onset diabetes mellitus in older adults: the cardiovascular health study. Archives of internal medicine 169: 798–807. 10.1001/archinternmed.2009.21 19398692 PMC2828342

[6] Knoops KT, de Groot LC, Kromhout D, Perrin AE, Moreiras-Varela O, et al (2004) Mediterranean diet, lifestyle factors, and 10-year mortality in elderly European men and women: the HALE project. JAMA 292: 1433–1439. 15383513 10.1001/jama.292.12.1433

[7] Laaksonen M, Prattala R, Karisto A (2001) Patterns of unhealthy behaviour in Finland. Eur J Public Health 11: 294–300. 11582610 10.1093/eurpub/11.3.294

[8] Tobias M, Jackson G, Yeh LC, Huang K (2007) Do healthy and unhealthy behaviours cluster in New Zealand? Australian and New Zealand journal of public health 31: 155–163. 17461007 10.1111/j.1753-6405.2007.00034.x

[9] Batty GD, Kivimaki M, Gray L, Smith GD, Marmot MG, et al (2008) Cigarette smoking and site-specific cancer mortality: testing uncertain associations using extended follow-up of the original Whitehall study. Annals of oncology: official journal of the European Society for Medical Oncology / ESMO 19: 996–1002. 10.1093/annonc/mdm578 18212091

[10] de Vries H, van’t Riet J, Spigt M, Metsemakers J, van den Akker M, et al (2008) Clusters of lifestyle behaviors: results from the Dutch SMILE study. Prev Med 46: 203–208. 17904212 10.1016/j.ypmed.2007.08.005

[11] Djousse L, Driver JA, Gaziano JM (2009) Relation between modifiable lifestyle factors and lifetime risk of heart failure. JAMA: J Am Med Assoc 302: 394–400. 10.1001/jama.2009.1062 PMC2742484 19622818

[12] Forman JP, Stampfer MJ, Curhan GC (2009) Diet and lifestyle risk factors associated with incident hypertension in women. JAMA: J Am Med Assoc 302: 401–411. 10.1001/jama.2009.1060 PMC2803081 19622819

[13] Eguchi E, Iso H, Tanabe N, Wada Y, Yatsuya H, et al (2012) Healthy lifestyle behaviours and cardiovascular mortality among Japanese men and women: the Japan collaborative cohort study. Eur Heart J 33: 467–477. 10.1093/eurheartj/ehr429 22334626

[14] Odegaard AO, Koh WP, Gross MD, Yuan JM, Pereira MA (2011) Combined lifestyle factors and cardiovascular disease mortality in Chinese men and women: the Singapore Chinese health study. Circulation 124: 2847–2854. 10.1161/CIRCULATIONAHA.111.048843 22104554 PMC3400937

[15] Khaw KT, Wareham N, Bingham S, Welch A, Luben R, et al (2008) Combined impact of health behaviours and mortality in men and women: the EPIC-Norfolk prospective population study. PLoS Medicine 5: e12 10.1371/journal.pmed.0050012 18184033 PMC2174962

[16] Mou J, Griffiths SM, Fong H, Dawes MG (2013) Health of China’s rural-urban migrants and their families: a review of literature from 2000 to 2012. Br Med Bull. 106:19–43. 10.1093/bmb/ldt016 23690451

[17] Cui X, Rockett IR, Yang T, Cao R (2012) Work stress, life stress, and smoking among rural-urban migrant workers in China. BMC Public Health 12: 979 10.1186/1471-2458-12-979 23151299 PMC3584974

[18] Li L, Wang HM, Ye XJ, Jiang MM, Lou QY, et al (2007) The mental health status of Chinese rural-urban migrant workers: comparison with permanent urban and rural dwellers. Soc Psychiatry Psychiatr Epidemiol 42: 716–722. 17598056 10.1007/s00127-007-0221-0

[19] Zhong BL, Liu TB, Chiu HF, Chan SS, Hu CY, et al (2013) Prevalence of psychological symptoms in contemporary Chinese rural-to-urban migrant workers: an exploratory meta-analysis of observational studies using the SCL-90-R. Soc Psychiatry Psychiatr Epidemiol 48(10):1569–1581. 10.1007/s00127-013-0672-4 23508367

[20] Zhu CY, Wang JJ, Fu XH, Zhou ZH, Zhao J, et al (2012) Correlates of quality of life in China rural-urban female migrate workers. Qual Life Res 21:495–503. 10.1007/s11136-011-9950-3 21695594

[21] Jiang S, Zhang L, Wang W (2007) The mental health of migrant workers in Chongqing City. Psychol Sci 30: 216–218.

[22] Shanghai government affairs (2011) 8,977,000 of foreign resident population in Shanghai, and growth of 193.6% in five years. Available: http://shzw.eastday.com/shzw/G/20110926/userobject1ai60714.html

[23] Best paper award (2008) 315-Shanghai migrant workers survey report in 2007. Available: http://yxlw.acftu.org/template/6/file.jsp?cid=15&aid=230

[24] Shanghai statistics (2013) Marital and birth status of our city’s migrant workers. Available: http://www.stats-sh.gov.cn/fxbg/201307/258527.html.

[25] Shanghai statistics (2013) Living conditions of our city’s migrant workers. Available: http://www.stats-sh.gov.cn/fxbg/201306/258477.html

[26] Shanghai statistics (2013) Participating in the social security situation of our city’s migrant workers. Available: http://www.stats-sh.gov.cn/fxbg/201307/258596.html.

[27] Shanghai statistics (2008) More than 400 million migrant workers entered Shanghai in 2007. Available:http://www.stats-sh.gov.cn/fxbg/201103/92223.html.

[28] The central peoples government of the peoples republic of China (2012) Shanghai adjust the minimum income and minimum living standards Available: http://www.gov.cn/fwxx/sh/2012-03/26/content_2100302.htm

[29] Shanghai statistics (2013) Urban residents per capita income and expenditure in 2012. Available: http://www.stats-sh.gov.cn/sjfb/201301/251782.html

[30] Wang ZY (1984) Symptom Check-list 90. Shanghai Archives of Psychiatry: 68–70.

[31] Jin H, Wu W, Zhang M (1986) Preliminary analysis of SCL-90assessment results in China populations. Chinese Journal of Nervous and Mental Diseases 12: 260–263.

[32] Zhang L, Zhao J, Xiao H, Zheng H, Xiao Y, et al (2014) Mental health and burnout in primary and secondary school teachers in the remote mountain areas of Guangdong Province in the People’s Republic of China. Neuropsychiatr Dis Treat 10:123–130. 10.2147/NDT.S56020 24465129 PMC3900332

[33] Chen F, Wang L, Heeramun-Aubeeluck A, Wang J, Shi J, et al (2014) Identification and characterization of college students with attenuated psychosis syndrome in China. Psychiatry Res 216: 346–350. 10.1016/j.psychres.2014.01.051 24636247

[34] Ministry of health disease Control department of the people’s Republic of China (2003) Chinese adult overweight and obesity prevention and control guidelines (Trial).

[35] Chen X, Li X, Stanton B, Fang X, Lin D, et al (2004) Cigarette smoking among rural-to-urban migrants in Beijing, China. Prev Med 39: 666–673. 15351531 10.1016/j.ypmed.2004.02.033

[36] Yang T, Wu J, Rockett IR, Abdullah AS, Beard J, et al (2009) Smoking patterns among Chinese rural-urban migrant workers. Public health 123: 743–749. 10.1016/j.puhe.2009.09.021 19896682

[37] Rubinsky AD, Dawson DA, Williams EC, Kivlahan DR, Bradley KA (2013) AUDIT-C Scores as a Scaled Marker of Mean Daily Drinking, Alcohol Use Disorder Severity, and Probability of Alcohol Dependence in a U.S. General Population Sample of Drinkers. Alcohol Clin Exp Res 8: 1380–1390. 10.1111/acer.12092 23906469

[38] Bradley KA, Kivlahan DR, Williams EC (2009) Brief approaches to alcohol screening: practical alternatives for primary care. J Gen Intern Med 24: 881–883. 10.1007/s11606-009-1014-9 19495888 PMC2695519

[39] Reinert DF, Allen JP (2007) The alcohol use disorders identification test: an update of research findings. Alcohol Clin Exp Res 31:185–99. 17250609 10.1111/j.1530-0277.2006.00295.x

[40] Cappuccio FP, D’Elia L, Strazzullo P, Miller MA (2010) Sleep duration and all-cause mortality: a systematic review and meta-analysis of prospective studies. Sleep 33: 585–592. 20469800 10.1093/sleep/33.5.585 PMC2864873

[41] Chinese Nutrition Society: The dietary guidelines for Chinese residents (2008) Beijing: Tibert People Publishing House.

[42] Kvaavik E, Batty GD, Ursin G, Huxley R, Gale CR (2010) Influence of individual and combined health behaviors on total and cause-specific mortality in men and women: the United Kingdom health and lifestyle survey. Archives of internal medicine 170: 711–718. 10.1001/archinternmed.2010.76 20421558

[43] van Dam RM, Li T, Spiegelman D, Franco OH, Hu FB (2008) Combined impact of lifestyle factors on mortality: prospective cohort study in US women. BMJ 337: a1440 10.1136/bmj.a1440 18796495 PMC2658866

[44] Carlsson AC, Wändell PE, Gigante B, Leander K, Hellenius ML, et al (2013) Seven modifiable lifestyle factors predict reduced risk for ischemic cardiovascular disease and all-causemortality regardless of body mass index: a cohort study. Int J Cardiol 168: 946–952. 10.1016/j.ijcard.2012.10.045 23181992

[45] The central peoples government of the peoples republic of China (2013) National migrant workers monitoring report. Available: http://www.gov.cn/xinwen/2014-05/12/content_2677889.htm

[46] Haiyan Xing (2008) The research of health status of migrant workers and related health policy. Zhejiang University:

[47] Chinese center for disease control and prevention (2013) China Cardiovascular Disease Report 2012” released-One person dies of cardiovascular disease each 10 seconds. Available: http://www.chinacdc.cn/mtdx/mxfcrxjbxx/201308/t20130813_86592.htm

[48] Gao Y, Chen G, Tian H, Lin L, Lu J, et al (2013) Prevalence of hypertension in china: a cross-sectional study. PLoS One 8: e65938 10.1371/journal.pone.0065938 23776574 PMC3679057

[49] Prevention Ccfdca (2011) 2010 national disease surveillance points (DSPs) the main surveillance result of chronic disease and risk factors. Available: http://www.chinacdc.cn/gwswxx/mbsqc/201109/t20110906_52141.htm.

[50] Singh GK, Siahpush M, Hiatt RA, Timsina LR (2011) Dramatic increases in obesity and overweight prevalence and body mass index among ethnic-immigrant and social class groups in the United States, 1976–2008. Journal of community health 36: 94–110. 10.1007/s10900-010-9287-9 20549318 PMC3020302

[51] Wang Y, Beydoun MA (2007) The obesity epidemic in the United States—gender, age, socioeconomic, racial/ethnic, and geographic characteristics: a systematic review and meta-regression analysis. Epidemiologic reviews 29: 6–28. 17510091 10.1093/epirev/mxm007

[52] Yang T, Xu X, Li M, Rockett IR, Zhu W, et al (2012) Mental health status and related characteristics of Chinese male rural—urban migrant workers. Community Ment Hit J 48:342–351. 10.1007/s10597-011-9395-8 21394472

[53] Li X, Stanton B, Fang X, Lin D (2006) Social stigma and mental health among rural-to-urban migrants in China: a conceptual framework and future research needs. World Health Popul 8: 14–31 18277106 10.12927/whp.2006.18282 PMC2249560

[54] Hesketh T, Lu L, Jun YX, Mei WH (2007) Smoking, cessation and expenditure in low income Chinese: cross sectional survey. BMC public health 7: 29 17335587 10.1186/1471-2458-7-29 PMC1821015

[55] Tsai SY (2012) A study of the health-related quality of life and work-related stress of white-collar migrant workers. International journal of environmental research and public health 9: 3740–3754. 10.3390/ijerph9103740 23202771 PMC3509477

[56] GuanSheng M, SongMing D, LiNan H, YanPing L, XiaoQi H, et al (2009) The prevalence of heavy drinking among adults in China. Acta Nutrimenta Sinica 31: 213–217.

[57] Ma Y, Zhang B, Wang H, Du W, Su C, et al (2011) The Effect of Alcohol Consumption on Prevalence of Hypertension among Adults Residents from 9 Provinces of China. Chinese Journal of Prevention and Control of Chronic Diseases 19: 9–12.

[58] Piwonski J, Piwonska A, Sygnowska E (2010) Do depressive symptoms adversely affect the lifestyle? Results of the WOBASZ study. Kardiologia polska 68: 912–918. 20730723

[59] Prochaska JJ, Spring B, Nigg CR (2008) Multiple health behavior change research: an introduction and overview. Preventive medicine 46: 181–188. 10.1016/j.ypmed.2008.02.001 18319098 PMC2288583

[60] Shi Y, Ji Y, Sun J, Wang Y, Sun X, et al (2012) Lack of health risk awareness in low-income Chinese youth migrants: assessment and associated factors. Environ Health Prev Med 17:385–393. 22286867 10.1007/s12199-012-0264-z PMC3437355

[61] Li Y (2013) Understanding health constraints among rural-to-urban migrants in China. Qual Health Res 23:1459–1469. 10.1177/1049732313507500 24122513

[62] Lin D, Li X, Wang B, Hong Y, Fang X, et al (2011) Discrimination, perceived social inequity, and mental health among rural-to-urban migrants in China. Community Ment Health J 47:171–180. 10.1007/s10597-009-9278-4 20033772 PMC2891847

[63] Hesketh T, Ye XJ, Li L, Wang HM (2008) Health status and access to health care of migrant workers in China. Public Health Rep 123:189–197. 18457071 10.1177/003335490812300211 PMC2239328

